# Candidiasis in Pregnancy: Relevant Aspects of the Pathology for the Mother and the Fetus and Therapeutic Strategies

**DOI:** 10.3390/tropicalmed9050114

**Published:** 2024-05-15

**Authors:** Alessandro Messina, Alessia Mariani, Romina Brandolisio, Elena Tavella, Chiara Germano, Giovanni Lipari, Livio Leo, Bianca Masturzo, Paolo Manzoni

**Affiliations:** 1 Division of Obstetrics and Gynecology, Department of Maternal, Neonatal and Infant Medicine, University Hospital “Degli Infermi”, 13875 Ponderano, Italyalessia.mariani@aslbi.piemonte.it (A.M.); chiara.germano@aslbi.piemonte.it (C.G.); giovanni.lipari@aslbi.piemonte.it (G.L.); bianca.masturzo@aslbi.piemonte.it (B.M.); 2 Division of Pediatrics and Neonatology, Department of Maternal, Neonatal and Infant Medicine, University Hospital “Degli Infermi”, 13875 Ponderano, Italy; lilianromina.brandolisio@aslbi.piemonte.it (R.B.); ele.tavella@gmail.com (E.T.); 3 Department of Maternal, Neonatal and Infant Medicine, University of Torino School of Medicine, 10125 Turin, Italy; 4 Division of Obstetrics and Gynecology, Hopital Beauregard, AUSL Valleè d’Aoste, 11100 Aosta, Italy; lleo@ausl.vda.it

**Keywords:** candida, candidiasis, pregnancy, transmission, newborn, treatment outcome

## Abstract

Vulvovaginal candidiasis (VVC) is a common condition that can lead to significant discomfort, affecting approximately 70–75% of women at least once in their lives. During pregnancy, the prevalence of VVC is estimated to be around 20%, peaking at about 30% in the third trimester, with a number of specific risk factors predisposing to yeast infection being identified and needing elucidation. This review aims to provide updated knowledge on candidiasis during pregnancy, addressing risk factors and maternal and neonatal outcomes, as well as discussing optimal therapeutic strategies to safeguard mothers and newborns. The bibliographic search involved two biomedical databases, PubMed and Embase, without imposing time limits. Among all *Candida* spp., *Candida albicans* remains the most frequent causative species. The hyperestrogenic environment of the vaginal mucosa and reduced immune defenses, physiological effects of pregnancy, create conditions favorable for *Candida* spp. vaginal colonization and hence VVC. Recent evidence shows an association between VVC and adverse obstetric outcomes, including premature membrane rupture (PROM), chorioamnionitis, preterm birth, and puerperal infections. Prompt and effective management of this condition is therefore crucial to prevent adverse obstetric outcomes, maternal–fetal transmission, and neonatal disease. Additional studies are required to confirm the benefits of systemic treatment for maternal candida infection or colonization in preventing premature birth or neonatal systemic candidiasis.

## 1. Introduction

Vulvovaginal candidiasis (VVC) is an infection that affects the vagina and vaginal vestibule, extending to the labia (minora and majora) and potentially the perianal region; candidiasis at the cervical or endometrial level is not documented in the literature. *Candida* spp. can colonize the vagina asymptomatically or lead to various symptoms, including pruritus, vaginal discharge (such as soreness, swelling, dyspareunia, dysuria), and increased discharge [1]. VVC can be diagnosed clinically, through microscopy, or with yeast culture. While the latter has traditionally been considered the gold standard for diagnosing vaginal fungal infections, it is worth noting that fewer than half of patients treated for VVC are diagnosed using this objective assay.

Recurrent VVC is defined as experiencing three or more symptomatic episodes of VVC within a 12-month period [1]. About 138 million women globally suffer from recurrent VVC each year [2].

VVC causes significant discomfort [3], with approximately 70% of women experiencing VVC at least once in their lifetime [4].

The main predisposing factor to yeast infection in the vaginal domain is the use of antimicrobial medications, which disrupt the beneficial *Lactobacillus* spp. microflora of the normal vaginal microbiota. Other common predisposing factors include diabetes, estrogens, immunosuppressive treatments and immunodeficiency conditions, genetic predispositions (genetic polymorphisms), the use of glucocorticoids, oral contraceptives, hormone replacement therapy, psychosocial stress, and sexual activity. However, a specific trigger factor is frequently not identified [5,6,7].

*Candida albicans* and other *Candida* spp. are known to have the ability to produce biofilms, hence taking advantage of the protective slime that allows *Candida* spp. to adhere firmly to mucosal surfaces and prosthetic devices, to remain nested in a confined environment where many systemic antifungal drugs cannot penetrate, and hence to maintain infection, particularly in immunocompromised patients. Two different types of biofilms can be involved in vaginal candidiasis: abiotic biofilms that require a plastic or metal substrate, such as intrauterine devices, and biotic biofilms that utilize the vaginal epithelium as the supporting base [8,9]. Biotic biofilms provide a favorable environment for the formation of persister cells, which prove to be highly resistant to first-line antifungals [10]. A recent study has shown how some strains (biofilm-positive) have indeed caused histopathological damage and inflammation, thus supporting the hypothesis that the biofilm plays an important role in the histopathogenesis of vaginal candidiasis [11].

During acute infection, inflammasome receptors play a fundamental role. In the vaginal epithelium, these types of receptors are activated by inflammatory factors and fungal components, such as glucan, chitin, and mannan, that stimulate several inflammatory cytokines by binding to specific macrophage receptors [12]. Consequently, these mechanisms lead to the activation of innate immunity and inflammasomes, particularly NLRP3, which play a crucial role in the pathogenesis of VVC [13]. Fungi, such as *Candida* spp., contain cytoplasmic estrogen receptors, and their activation facilitates the transition from yeast to hyphal form [14]. The virulence and pathogenicity of *Candida* spp. increase upon activation, explaining why women of childbearing age have an increased risk of developing VVC [8].

Proposed virulence determinants of *Candida albicans* involved in the pathogenesis of vaginal candidiasis include fungal morphogenesis, adhesion to vaginal epithelial cells, the production of phospholipases and proteinases such as secreted aspartyl proteases (Saps), and the presence of candidalysin, a well-identified secreted cytolytic peptide toxin [15]. This toxin represents one of the main virulence factors of *Candida* spp. along with proteases and lipases. Candidalysin plays a role in inducing infection starting from the yeast form, while granulocytes, formed as a result of the presence of pseudohyphae, are responsible for inflammation [16,17,18]. Before being secreted, candidalysin is incorporated into a polyprotein precursor called Ece1. Ece1 comprises a secretion signal peptide, the precursor peptide for candidalysin, and seven additional Ece1 peptides. This structural arrangement is probably necessary to prevent autoaggregation due to the amphipathic and hydrophobic nature of the candidalysin peptide [19]. Indeed, synthetic candidalysin tends to spontaneously form aggregates in aqueous solution [20]. The immune response induced by candidalysin during VVC is not protective [21]: despite the recruitment of large numbers of neutrophils, they do not promote fungal clearance [22]. This dysfunctionality has been attributed to specific host factors in the vaginal environment, including heparan sulfate, anti-*C. albicans* antibodies, and perinuclear anti-neutrophil cytoplasmic antibodies [23]. Neutralization of candidalysin or modulation of downstream inflammatory responses have been suggested as a therapeutic strategy to prevent immunopathology and symptoms during VVC [24].

Recent literature investigated the association between VVC and adverse obstetric outcomes, including premature membrane rupture (PROM), chorioamnionitis, preterm birth, and puerperal infections, as well as examining clues on maternal–fetal transmission, the onset of symptoms in the newborn, and the available treatment options.

The purpose of this review is, therefore, to strengthen the current knowledge regarding candidiasis in pregnancy and to discuss the best therapeutic strategies to safeguard the health of mothers and their newborns.

## 2. Materials and Methods

For the bibliographic search, two biomedical databases, PubMed and Embase, were consulted. The search strategy did not include time limits and utilized the following keywords: candida, candidiasis, pregnancy, transmission, newborn, treatment, and outcome.

## 3. Results

### 3.1. Candidiasis and Pregnancy

Vulvovaginal yeast infections during pregnancy are very frequent, and they can lead to significant inflammation, potentially contributing to adverse perinatal outcomes [25].

They are mostly attributed to candida species, and, generally, they are more prevalent in pregnant women compared to non-pregnant ones [26]. In fact, approximately 20% of women experience candidiasis during pregnancy, with this figure increasing up to nearly 30% in the third trimester of gestation [4]. Currently, it is not clear whether pregnant women carry higher levels of yeast organism loads compared to their non-pregnant counterparts, nor whether such levels are correlated with inflammation or may contribute to adverse perinatal outcomes [27].

Currently, it is well known that during pregnancy the vagina is colonized by *Candida* spp. in at least 20% of cases, and that this prevalence increases to 30% in immunocompromised patients, primarily due to elevated estrogen levels [4]. *Candida albicans* is the predominant species in most cases, followed by non-albicans species such as *Candida glabrata*, *Candida tropicalis*, *Candida krusei*, and *Candida parapsilosis*. Non-albicans species may result in milder symptoms compared to those caused by *Candida albicans* [28], and, in the etiopathogenesis of this condition, the importance of host factors becomes evident, especially during the transition from colonization to vaginitis. Indeed, the hyperestrogenic environment of the vaginal mucosa, combined with increased vaginal glycogen and a physiological reduction in immune defenses, enhances the colonization of *Candida* spp. [29]. Estrogen levels promote the transition of *Candida* spp. from the yeast form to the invasive filamentous form, facilitating the production of a peptide known as candidalysin [22], which is a toxin peptide of *Candida albicans* that exerts a cytotoxic effect on host cells, promotes invasion, recruits leukocytes, and stimulates nonspecific defenses against infection.

Additionally, mannoproteins enable *Candida* spp. to adhere to the vaginal epithelial surface, while aspartate secretory proteinases play a role in protein hydrolysis. All this interplay contributes to the pathogenic mechanisms of candida infections during pregnancy.

In addition, it is well noted that a hyperglycemic environment may favor establishment, adhesion, and proliferation of *Candida* spp. in tissues, vessels, and mucosal surfaces [30]; in fact, there is preclinical evidence that glucose enhances the expression of C3 fungal receptors, that biofilm growth of several pathogens, including *Candida* spp., has a threshold response to glucose in vitro, and that there is a substantial increase in biofilm production beyond a glucose concentration threshold of 200 to 240 mg/dL [31].

Under hyperglycemic conditions, the growth and adhesion of fungi increase, compromising the migration of neutrophils, chemotaxis, and phagocytosis [6].

Recent data show that systemic colonization by *Candida* spp. in preterm neonates occurs three times more frequently under hyperglycemic conditions and that systemic candida infection in such patients also increases when glycemic control is not stable [32].

Owing to this evidence, it might be reasonable to expect that women with gestational diabetes mellitus (GDM) are at higher risk and more likely to develop candida infections [33].

However, it is not entirely clear whether GDM confers a higher risk of developing VVC and the VCC-associated adverse obstetric outcomes, including premature rupture of membranes (PROM), preterm birth, chorioamnionitis, and even puerperal infections [34].

According to a number of reports, vulvovaginal yeast infections during pregnancy are mostly associated with multiparity, high level of education, and chronic use of antibiotics [35]. In this regard, host factors and intrauterine inflammation seem to be part of a general pathophysiological mechanism leading to various adverse pregnancy outcomes, such as premature rupture of the membranes, a significant precursor to preterm birth. Finally, data in the literature underline how, in many cases, the cause can be attributed to both intrauterine inflammation and microbial invasion of the amniotic cavity [36], and that in the case of some less common conditions, such as congenital fetal candidiasis and candida amnionitis, transmission during labor and vaginal delivery is more likely [37].

### 3.2. Candidiasis in Pregnancy and Its Impact on the Newborn

#### 3.2.1. Preterm Birth

The first month of life is the most vulnerable period for child survival, with 2.3 million newborns dying in 2022. Although neonatal mortality has decreased by 44% in the last 20 years, as of the year 2022 nearly half (47%) of all deaths in children under 5 years of age still occur in the first 28 days of life [38]. In this regard, adverse pregnancy outcomes, including preterm birth, pose significant obstetrical and public health challenges. Although the causes of preterm birth are often unknown, intrauterine infection plays a major role, accounting for a causative relationship in up 40% of cases. Of note, the most frequent pathway to intrauterine infection is ascending genital tract infection [39], including VVC.

There is a growing body of evidence suggesting that genital tract infection or inflammation, along with placental malperfusion syndromes, could be an underlying factor for both preterm birth and fetal growth restriction [40].

Preterm birth may occur spontaneously, such as following premature rupture of the amniotic membranes before labor, or it may be induced following a clinical decision subsequent to the detection of complications. The premature newborn presents a series of potential risks to various organ systems, including the central nervous system, lungs, and cardiovascular system. Individuals born preterm face a higher risk of mortality compared to those born full term, with a doubled risk of death particularly in young adults born extremely preterm (before 28 weeks gestation) [41].

The first trimester of pregnancy is a vulnerable period for the development of inflammatory responses associated with infections, which could serve as the triggering factor for preterm birth. The microorganisms present in the female genital tract can have pathogenic effects during pregnancy through infection of the amniotic cavity and/or by stimulating inflammatory cascades [42]. In addition to prostaglandins, chemokines and pro-inflammatory cytokines can contribute to cervical maturation and induction of contractions [43].

It is known that up to 40% of preterm births are attributed to bacterial or fungal vaginal infections [36]. Nevertheless, a recent meta-analysis including more than 34,700 women did not document a specific association between vaginal candida infection and preterm birth [44] However, *Candida albicans* has been identified in amniotic fluid samples from cases of spontaneous preterm birth and is also associated with fetal death and suboptimal neurological development [45]. The debate about yeast infections of the female genitalia and their association with preterm birth remains, therefore, current and open, with chronic inflammation following VVC being speculated as the true trigger for preterm birth. In women with VVC, an increase in the levels of inflammatory mediators in vaginal fluid, including interleukin [13], is documented [46], with symptomatic yeast infections being likely to cause a higher inflammatory response than asymptomatic infection [47]. Owing to the above evidence and considerations, it seems reasonable to make all possible efforts to prevent VVC, with some evidence suggesting that these strategies might actually impact and ultimately be linked to a lower incidence of preterm birth [48].

#### 3.2.2. Fetal Growth Restriction

Intrauterine inflammation appears to be a pathophysiological mechanism underlying a range of adverse pregnancy outcomes, including fetal growth restriction. This condition is associated with increased maternal systemic inflammation, elevated levels of cytokines, and proinflammatory markers [49].

Reducing local inflammation through the use of antifungal agents appears to lead to the reduction of a number of adverse outcomes, including fetal growth restriction. The underlying mechanism is speculated to involve reduced neutrophil attraction to vaginal epithelial cells (in vitro) and a reduction in production of inflammatory cytokines by these cells [50].

#### 3.2.3. Vertical and Horizontal Transmission

**Figure 1:**
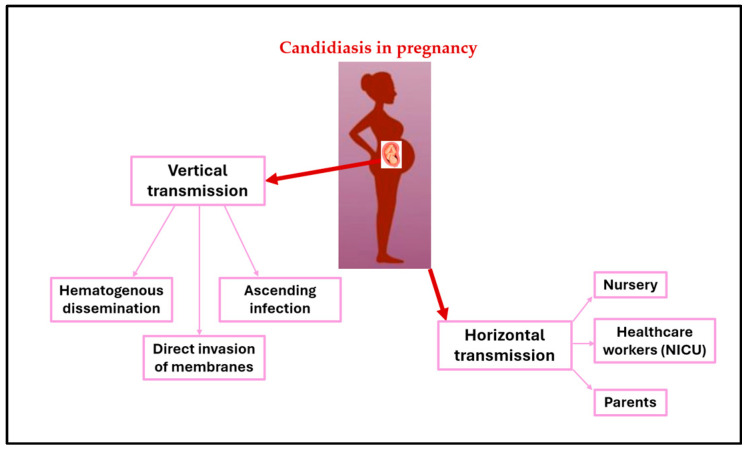
Vertical and horizontal transmission of Candida.

Neonates can acquire *Candida* spp. colonization through either vertical transmission from the mother or horizontal (nosocomial) transmission (see Figure 1). In the case of horizontal transmission, neonates can acquire *Candida* spp. from the nursery, their parents, or, most often, from healthcare workers in the neonatal intensive care unit (NICU) [51]. Horizontal acquisition, especially in NICUs, is likely the most common mode of transmission for some specific strains of *Candida* spp., for example *Candida parapsilosis* [52].

The evidence indicates that nosocomial acquisition of *Candida* spp. leading to infection primarily occurs through contact with the hands of healthcare workers. *Candida* spp. have been found in up to 29% of healthcare workers, with *Candida parapsilosis* isolated in 19% of cases and *Candida albicans* in 5% from hand skin cultures [53]. Horizontal acquisition of *Candida* spp. and subsequent colonization may progress to systemic invasion in up to 10% of colonized neonates, particularly in the most premature ones or those with underlying comorbidities [54]. The progression of colonization can result in dissemination and consequently late-onset invasive fungal infection.

Vertical transmission of *Candida* spp. from a mother who has been colonized or affected by *Candida* spp. during pregnancy usually involves the transmission of Candida strains to the newborn during the perinatal period via the genitourinary or gastrointestinal tract [55]. Studies using molecular typing techniques have shown that vertical transmission of *Candida albicans* has been detected in 33% of preterm infants born to mothers with VVC [56]. When Candida chorioamnionitis occurs, it can lead to intrauterine fetal death or preterm delivery. Mechanisms for intrauterine infection include hematogenous dissemination from mother to fetus, direct invasion of membranes, and ascending infection after spontaneous rupture of membranes. Vertical transmission of *Candida* spp. may result in severe forms of early-onset systemic infection in the neonate, with cutaneous congenital candidiasis (CCC) being a common clinical feature [57,58,59].

### 3.3. Congenital Cutaneous Candidiasis

Congenital cutaneous candidiasis (CCC) is indeed an extremely rare condition that can occur in both full-term and preterm infants. A meta-analysis conducted in 2020 revealed that fewer than 50 recognized cases had been reported in the literature over the past 54 years. This rarity emphasizes the unique and uncommon nature of CCC [44].

CCC can occur when the fetus or neonate comes into contact with colonized or infected maternal fluids or mucosa either in utero or during passage through the birth canal. This contact during the perinatal period can lead to the transmission of *Candida* spp. from the mother to the newborn, potentially resulting in systemic infection [47,55,59,60].

Risk factors for CCC include VVC, prolonged rupture of membranes, and the presence of an intrauterine foreign body, such as a cerclage suture. The actual incidence of CCC is debated, as it may occur more frequently than generally thought. Due to a favorable outcome in many cases, CCC can go unnoticed or be mistaken for other dermatoses appearing in the neonatal period, highlighting the need for a differential diagnosis with other newborn pathologies. Improved and accurate diagnosis of this form of systemic candidiasis could help address the existing gap in estimating the burden of vertically-acquired, symptomatic neonatal candidiasis [61].

CCC is triggered by an intrauterine infection through vaginal colonization by *Candida albicans*, which is present during pregnancy in 20–30% of cases; however, only 1% of individuals develop congenital infection, explaining the low incidence of the disease. The rash typically appears within 36–72 h after birth, depending on the inoculum, number of organisms, and neonatal immune response [57,62,63].

Infants with CCC have an extensive maculopapular rash with massive desquamation, resembling staphylococcal scalded skin syndrome. Other findings include pustules, cracking skin, funisitis, diffuse erythema, desquamation, and placental infection with abscesses or yellow plaques or secretions [57,58,64]. Leukocytosis and fluid and electrolyte imbalances are common in premature newborns. Notably, in term infants, clinical symptoms and signs may occasionally be confined to the skin without systemic involvement. In preterm infants, however, this is frequently a life-threatening disease. When systemic compromise occurs, it always requires prompt and aggressive parenteral treatment. Early-onset candidemia can often be associated with end-organ dissemination and involvement (such as meningitis, bronchopneumonia, arthritis, and endocarditis), leading to very high mortality rates [57,59]. In such cases, *Candida albicans* is the most frequently found fungal pathogen, and it can be isolated in blood, urine, cerebrospinal fluid (CSF), and skin culture [57,59].

The treatment of CCC involves administering amphotericin B deoxycholate intravenously at a dosage of 1 mg/kg daily for initial therapy. Fluconazole may be utilized for isolates susceptible to fluconazole, with a loading dose of 25 mg/kg followed by 12 mg/kg daily, on the condition that neither the mother nor the infant has had prior exposure to this azole. The systemic treatment duration should not be less than 20 days, and in all cases it should extend for at least an additional two weeks following clearance of documented blood culture and local cultures [59].

### 3.4. Treatment Lines for VVC in Pregnancy

Strategies aimed at treating VVC in pregnancy need obviously to cope with the risk of teratogenesis attributable to several classes of medications during such a vulnerable period, and this is why clearcut recommendations for specific antifungal treatment in gestational VVC are lacking.

Although some authors have reported a decrease in the rate of preterm birth after vaginal treatment with clotrimazole in VVC cases where treatment had been instituted early during gestation, e.g., in the first trimester [65,66], most of the published evidence suggests that a specific antifungal treatment should be considered as not strictly necessary when asymptomatic Candida colonization and VVC are detected during the first and second trimesters. In turn, at late gestational epochs it is crucial to administer prophylactic antifungal treatment in the last week of pregnancy to prevent transmission during delivery, when VVC has its onset and/or is identified in the third trimester [4], even in instances of asymptomatic colonization during the last week of pregnancy, owing to the risk of occurrence of a rare, but dramatic condition such as CCC, as previously discussed.

This strategy appears effective in preventing transmission to the newborn during vaginal birth, with a substantial decrease of the incidence of oral thrush and diaper rash in the newborn baby, reducing their occurrence from 10% to 2% in the first four weeks of life [67].

Regarding therapy in cases of symptomatic disease, the primary treatment for symptomatic vaginitis during pregnancy is vaginal clotrimazole, with a dosage of 500 mg for up to seven days [4,30]. This is particularly recommended during the first trimester due to the observed increased risk of miscarriage [68] and fetal malformations associated with oral fluconazole, including conditions such as fetal cleft lip and transposition of the great vessels [69], and of Fallot’s tetralogy [70]. In instances of asymptomatic colonization detected during the first and second trimesters, clotrimazole (and, more generally, pharmacological) treatment is not deemed necessary.

If VVC is identified in the third trimester, it is imperative to administer prophylactic antifungal treatment in the last week of pregnancy.

Oral fluconazole doses ranging from 150 to 300 mg have been deemed safe, starting in the second trimester of gestation, although fluconazole has not received official approval for use during pregnancy [30].

Promising recent data suggest that targeting candidalysin through innovative therapies could interrupt the hyperinflammatory loop that drives VVC immunopathology and the severity of symptoms [71].

### 3.5. Role of Probiotics in the Treatment of Candidiasis in Pregnancy

As mentioned earlier, supplementing the microbiota with lactobacilli is crucial in supporting medical treatment, given their capacity to produce a biofilm that inhibits pathogen growth. Among all lactobacilli strains, *Lactobacillus rhamnosus* is the one with the strongest evidence of beneficial effects in VVC. This probiotic enhances the production of a biofilm that inhibits the adhesion and growth of pathogens to the mucosal vaginal surfaces. In addition to oral probiotic administration, intramuscular delivery of non-H_2_O_2_-producing lactobacilli, eliciting a nonspecific immune response, has demonstrated promising results [72].

As studied in vitro, lactobacilli exert a direct antifungal and immunostimulating effect [73,74] and may inhibit the adhesion of pathogenic microorganisms and modulate the inflammatory response, enhancing the action of antifungal drugs.

Furthermore, in vivo they also appear to significantly reduce fungal colonization in VVC undergoing antifungal treatment [75].

Supplementation of lactobacilli for 6 days every month, together with a single dose of itraconazole, has been described by some authors as not improving VVC recurrence rates compared to itraconazole alone [76]. However, a recent study shows that probiotic lactobacilli are beneficial for pregnant women, especially in reducing vulvovaginal symptoms and VVC recurrences, which is accompanied by an improvement in emotional and social distress attributed to VVC [77]. Notably, *Lactobacillus plantarum* I1001, has proven useful in increasing the effectiveness of a single 500 mg dose of clotrimazole in preventing the recurrence of VVC [78].

Probiotics also have the ability to prevent the passage of pathogenic microbes from the gastrointestinal tract to the vagina, modulating the host’s immune response, enhancing epithelial defenses, and consequently impacting the expression of inflammatory genes induced by VVC [79]. Moreover, probiotics exhibit a direct fungicidal effect by preventing the adhesion of candida to epithelial cells [4,79].

In the light of all the above, the use of probiotics appears to be a beneficial adjunctive treatment due to its synergistic effect with conventional medical therapies in VVC.

Finally, lactoferrin, a glycoprotein present in milk and cervical mucus, has shown promising beneficial effects in some recent studies due to its ability to mitigate vaginal symptoms associated with candidiasis [80] and its synergistic antibiotic-like effect with *Lactobacillus rhamnosus*. Lactoferrin is recommended as a nutritional supplement at a dosage of 200 mg twice a day [81].

## 4. Discussion and Implications for Practice

Currently, screening for the detection of VVC in pregnancy is not included in international guidelines [4].

This is partly due to the point that asymptomatic candidiasis during pregnancy does not appear to be convincingly associated with the risk of preterm birth [63] and that treatment for asymptomatic colonization is not recommended unless it is in the third trimester of gestation, particularly in the last week before giving birth [4], as discussed in this review.

The cost/benefit ratio of candida screening during pregnancy for early detection of VVC remains, therefore, a partially unaddressed issue.

Several areas remain controversial and in need of further research and assessment.

First, identification of pregnant women at higher risk of developing symptomatic VVC is needed, as well as that of which test to perform, should a screening be carried out. In this regard, the use of molecular tests appears to be promising, both in terms of detection capability and quantification of organism. A recent multisite prospective cohort study demonstrated that the polymerase chain reaction for the candida group of pathogens (*C. albicans*, *C. dubliniensis*, *C. parapsilosis*, and *C. tropicalis*) exhibited high clinical accuracy, with a sensitivity of 90.9% and specificity of 94.1% [82]. Should these findings be further confirmed, molecular diagnostic methods could replace culture-based examination as the gold standard in diagnosing VVC. Hopefully, validation of an accurate test specifically designed for the pregnant population would not only allow for timely and targeted treatment of the infection, reducing the costs of misdiagnosis, but would also notably decrease the risks of transmission and inappropriate drug exposure for both the mother and the fetus.

Second, research should focus on understanding whether the presence of symptoms is associated with a higher organism burden and whether a higher organism burden is linked to a greater risk of mother/fetus transmission, and, ultimately, preterm birth. Better understanding the natural history of VVC during pregnancy could indeed help clinicians prevent the negative outcomes of VVC for both the mother and the newborn [47].

Finally, research on vaginal microbiota and the role of probiotics in prevention and management of VVC is needed, which also addresses the potential relationships among local disrupted microbiota, VVC, and concomitant vaginal or cervical infections.

## 5. Conclusions

VVC in pregnancy is a pathology that should not be underestimated, given its high prevalence, substantial discomfort for patients, and potential correlation with adverse obstetric outcomes affecting the newborn. It is crucial to promptly and effectively diagnose and (when appropriate) treat this condition.

Several areas of uncertainty remain in need of further elucidation, including the relationships between different timing of the onset of VVC during pregnancy and its short- and long-term severity, the cost/benefit ratio of instituting antifungal local and systemic treatment, the role of vaginal microbiota and of supplemented probiotics and lactoferrin, the correct timing for treating with local and/or systemic antifungals, the identification of high-risk women, the correct strategies to prevent candida transmission during labor and delivery, and the short- and long-term neonatal outcomes of maternal VVC.

## Data Availability

Since this is a review, no new data were generated or manipulated.
